# Analysis and design of terahertz reflectarrays based on graphene cell clusters

**DOI:** 10.1038/s41598-022-26382-w

**Published:** 2022-12-21

**Authors:** Parinaz Hosseini, Homayoon Oraizi

**Affiliations:** School of Electrical Engineering, Iran University of Science and Technology, Tehran, 1684613114 Iran

**Keywords:** Engineering, Electrical and electronic engineering

## Abstract

In this paper, the graphene cell-cluster is introduced, which is composed of an array of identical unit-cells placed in a geometrical configuration. Such graphene cell-clusters are then used for the realization of a reflectarray. To the best of our knowledge, identical unit-cells in a particular geometrical configuration have already been introduced, but the analytical formulas for this model have not been investigated so far. In this paper, the Fourier-optics and aperture field estimation methods are applied to investigate the effect of cell-cluster dimensions on the generation of specified far-field radiation patterns. Implementing cell-clusters in graphene reflectarrays and similar structures, and also applying the proposed formulas, lead to the simplicity of configuration and enhancing the design accuracy. First, the effect of cell-cluster dimensions on the reflectarray radiation pattern is investigated. Then, a reflectarray composed of graphene cell-clusters is designed. A new configuration of graphene unit-cell composed of two graphene layers is proposed, where a middle layer of metallic patch is inserted. In the common graphene unit-cells, the rate of amplitude variations is quite high and greatly depends on the variation of phase in the proposed unit-cell. However, the amplitude variation is quite smaller than the phase variations.

## Introduction

The Terahertz frequency range spans from 0.1 to 10 Terahertz (0.1–10 THz). It falls between the microwave and infrared frequency ranges. It is of high interest nowadays due to its exclusive characteristics. Since its wavelength is shorter than that of the microwave frequencies, it possesses higher rates of data transmission in the telecommunication systems^1^, it realizes higher precision radars^2^, it may be used for spectroscopy^3^, it may also be used as a substitute to X-rays due to its nondestructive properties in applications of security imaging^4^, imaging of antique and precious objects^5^, and biological applications, such as imaging of human body surface tissues^6^.

A typical system operating at THz frequencies may be composed of a Terahertz signal generator^7^, a sample or target for imaging, signal reception and detection^8^. A complete THz system for pulse spectroscopy is depicted in reference^9^. It uses mirrors and lenses for the concentration and bending of THz signal beams. For the design of such devices, various technologies are used, such as simple monolithic structures, dielectric lenses^10^, metallic mirrors^11^, or more complex configurations, such as transmitarrays^12^, and reflectarrays^13^ using all types of unit-cells. They possess some dynamic control ability and adjustability. These unit cells may be made up of adjustable liquid crystals^14^, graphene paches^15^, graphene and metal patches^16^, metasurfaces with electromagnetically induced transparency (EIT) technology^17^, and metasurfaces with plasma induced transparency (PIT) technology^18^.

Graphene is a two-dimensional crystalline matter composed of carbon atoms with exclusive properties, such as adjustability by an electrical voltage bias. The conductivity of graphene is obtained by the Kobu formula as

1 $$\sigma \left( \omega \right) = \frac{{ie^{2} E_{f} }}{{\pi \hbar^{2} \left( {\omega + i/\tau } \right)}} + \frac{{ie^{2} \omega }}{\pi }\mathop \smallint \limits_{0}^{\infty } \frac{{f\left( {\varepsilon - E_{f} } \right) - f\left( { - \varepsilon - E_{f} } \right)}}{{\left( {2\varepsilon } \right)^{2} - \left( {\hbar \omega + i\Gamma } \right)^{2} }}d\varepsilon .$$

where *e* is electron charge, *Ef* is Fermi energy level (depending on various parameters, such as impurity value, and electric voltage bias), ℏ is reduced Planck constant, *ω* is angular frequency, *τ* is relaxation time and *f* is Fermi-Dirac distribution function^19^. Reflectarrays are usually composed of single or multiple dielectric layers, on which resonant patches are placed. A metallic ground plane is fixed on its bottom side. The determination of the geometry and dimensions of each patch may dynamically adjust the characteristics of reflected waves. The graphene patches used as unit-cells with appropriate voltages may be used for such functions and purposes. For the determination of far-field radiation patterns of reflectarrays and antennas, numerical methods are commonly used, which are accurate and flexible^20–25^. In addition, analytical methods are effective to apply for the far-field synthesis^26–30^. Analytical methods and mathematical formulas are suitable for gaining an initial intuition of the relationship between the reflector structure and the far-field pattern. In the design of arrays and reflectors, analytical methods first provide an initial view of the overall structure to provide the desired pattern specifications. The numerical and optimization methods then are used to complete the design.

Graphene reflectarrays have been proposed since 2013 by Carrasco and Carrier^13^.The use of graphene unit-cells because the unique properties of graphene in the terahertz and infrared ranges have facilitated its applications. Their low plasmonic resistance losses and tunability with electrical biases are among their useful characteristics. In another paper^31^, Carrasco proposed a tunable graphene unit-cell structure with a variable voltage bias, in order to control the complex conductivity of graphene to adjust the phase of reflected field. Later on, graphene reflectarrays were designed to create vortex beams in the terahertz range^32–34^. Unit-cells consisting of simple square graphene patches with electrical tuning capability, were implemented to steer the vortex beam. Then, graphene reflectarrays with polarization dependent unit-cells were proposed. In^35^, rectangular graphene patches placed at different angular locations were used to change the phase of circularly polarized waves. In^36^, cross dipole graphene patches were implemented to convert the phase of the circularly polarized wave. In^37^, a new type of graphene unit-cell composed of graphene square patches and an epsilon-near-zero material, were proposed. A reflectarray operating in the THz band using this unit-cell was designed, using an epsilon-near-zero material with relative permittivity under 1. It helped to reduce the losses of graphene patch. In^38^, unit cells composed of graphene and metallic patches were proposed. In this structure, a crossed metallic dipole is placed inside a graphene square ring, in order to provide appropriate coupling for resonance. As a result, by using only 0.2 V chemical potential, the appropriate tunable phase in the range of 330° is achieved. In^39^, unit-cells with phoenix graphene patches are proposed for reflectarrrays to generate vortex beams in the THz range. In general, phoenix unit-cells are broadband structures. The use of such structural geometries for graphene patches provides the capability of phase variation equal to 360 degrees, unlike common graphene square unit cells. In^40^, the hexagonal graphene unit-cells were proposed. The reflectarray operating at 1.6 THz using this geometry was designed. Three different hexagonal-shaped graphene unit-cells designed in this paper, achieved the reflection phase in the range of 525 degrees.

The unit-cells composed of graphene are several times smaller than other types of unit-cells. Therefore, it is common to place a number of identical unit-cells in graphene reflectarrays. A trade-off should be done to select the best number of identical units. The details of this method, have not been addressed as yet. It is an important parameter in the design, which achieves suitable gain and simple structure.

In this paper, the concept of cell-clusters composed of a number of identical unit-cells, is presented. The tangential electric field on the reflectarray surface is considered as an aperture field distribution. The method of Fourier optics is used to obtain the relationship between the aperture field and the far-field radiation pattern. Consequently, the related mathematical formulas are proposed. Then, the optimum geometry of cell-clusters for the generation of specified radiation pattern is investigated. By the application of proposed method, the design process is simplified and optimized. Thereafter, to investigate the proposed method, a reflectarray for the generation of a flat-top pattern is designed. The dimensions of cell-clusters are changed to observe their effects on the far-field pattern. Finally, a reflectarray of graphene cell-clusters is designed and optimized for the generation of pencil-beams at various directions.

## The use of cell-cluster in the reflectarray design

### Definition of the graphene cell-cluster

Due to the plasmonic characteristics of graphene surfaces, the wavelength of excited wave on it is much shorter than the free space (*λ/*16) as noted in^41^. Therefore, for modulated graphene surfaces such as reflectors, dimensions of graphene unit-cells are also shorter than metallic ones. So, the surface impedance of a collection of small graphene cells arranged on a surface, may be considered as an equivalent surface impedance, which is considered to possess an average and equivalent impedance value. Consequently, the collection of small graphene unit-cells on a surface (as a cell-cluster) have small dimensions and will be characterized by an average surface impedance. For example, as proposed in^42^, by combining two different shapes of graphene unit-cells placed periodically in an array, larger values of surface impedance intervals may be achieved compared to a simple graphene unit-cell for the purpose of beam shaping.

**Figure 1:**
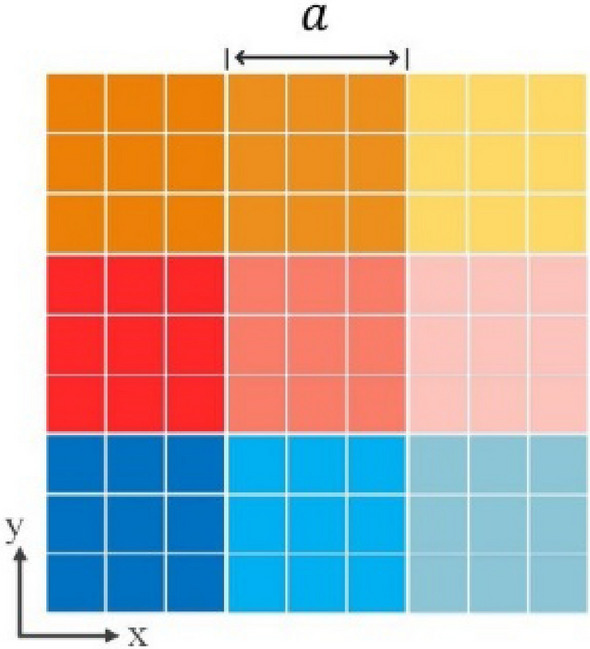
A structure composed of cell-clusters, each one consists of equivalent unit-cells shown with different colors.

Identical graphene unit-cells may be used in a cluster, which may provide several desired characteristics, such as reduction of phase error and simple construction in reconfigurable reflectarrays^43^. As shown in Fig. 1, each larger square composed of smaller squares of the same color, represents a cell-cluster composed of a collection of identical unit-cells. Each color represents a particular surface impedance, relevant to each cell-cluster.

### Investigatation of effect of cell-cluster dimensions using fourier optics

The Fourier optics technique is an effective analytical fmethod for estimating the far-zone pattern of a reflective metasurface. In the design of structures such as aperture antennas, the tangential field located on the radiating aperture^44^can be considered as a secondary source producing the far-field pattern, based on the Huygens theory^44^. This pattern is named the “aperture field distribution”. The related far-field pattern can be calculated theoretically by the Fourier optics method. For example, if the aperture field distribution is considered as *f*(*x, y*), the far-field pattern may be determined by the “Fourier transformation” technique as *F*(*u, v*), where *x* and *y* are the Cartesian coordinates on the aperture and where *u* = *k*_0_ sin *θ* cos*ϕ* and *v* = *k*_0_ sin *θ* sin *ϕ* indicate the variables in the related spectral coordinates. Generally, for the design of aperture antenna structures, the far-zone field can be defined as a specified objective field, and the aperture field distribution is an unknown pattern function to be determined. Consequently, it is an inverse problem.

In this paper for the design of reflectarray structures, we use the Fourier optics method, and similarly consider the tangential electric field as an aperture field distribution. In the proposed method, the tangential field distribution can be estimated relative to cell-clusters dimension, and the total reflectarray structure. So, the method can be termed as the *“aperture field estimation”*. The related aperture field distribution can be obtained from the desired far-field pattern. Then, the related structure composed of graphene cell-clusters can be designed.

For the Investigation of the effect of cell-cluster dimensions, this paper considers two separate functions as an aperture field distribution on the reflectarray. One function denoted as *f*(*x, y*) is the inverse Fourier transform, obtained from *F(u,v)* as the desired far-field pattern. The other function denoted as $e\left( {x,y} \right)$ represents a complete aperture field distribution on the reflectarray, related to cell-cluster configuration. This function more closely corresponds to the reflectarray structure, while *f*(*x, y*) is merely an estimation obtained from the desired far-field pattern. The function $e\left( {x,y} \right)$ may be obtained from *f*(*x, y*) mathematically by using the Fourier optics methods.

**Figure 2:**
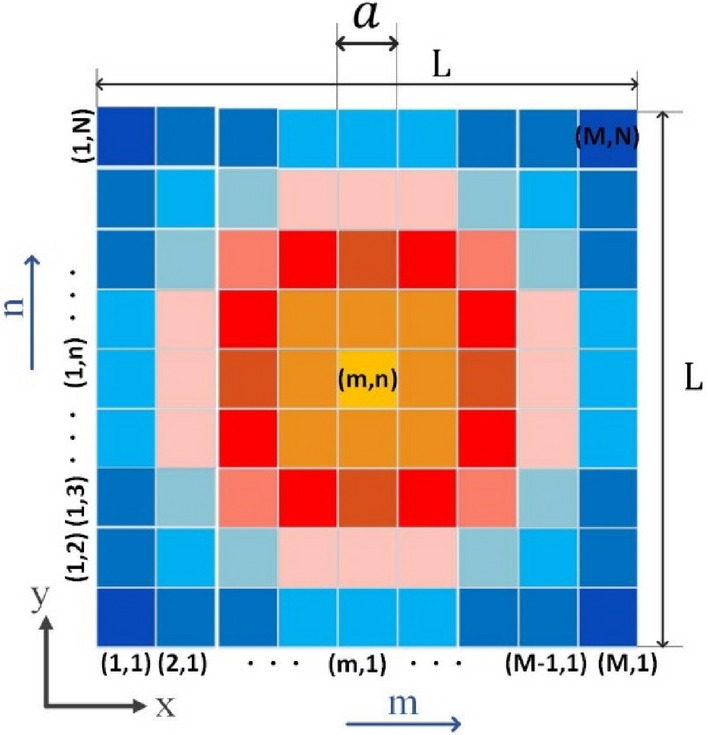
The conceptual schematic of a reflector surface consisting of cell-clusters.

**Figure 3:**
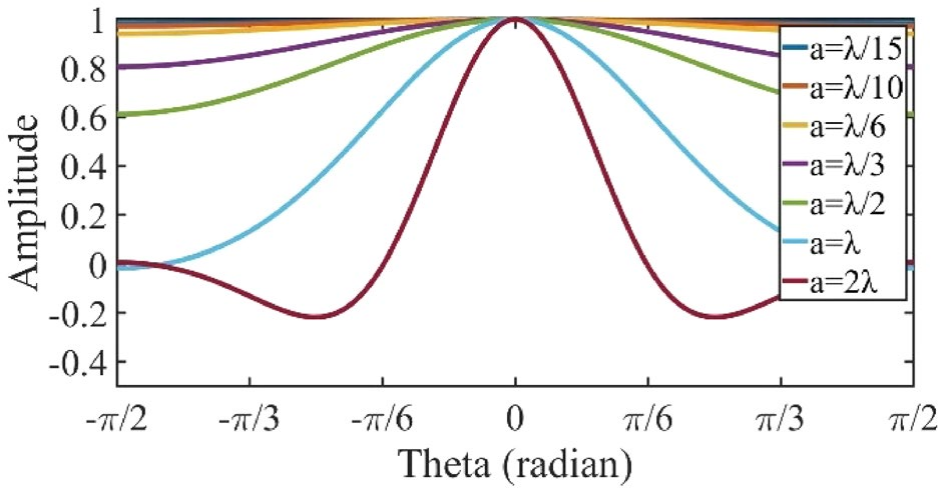
The magnitude of a one-dimensional *sinc* function for different values of *a*.

At first, in the reflectarray structure, the field distribution on each cell-cluster is considered to be ideally uniform (as indicated in Fig. 2). Then, an appropriate sampling should be performed on *f*(*x, y*) at specified points. In the simplest case, the sampling points can be chosen at the center of each cell-cluster. Note that, $\left( {x_{m} ,y_{n} } \right)$ indicates the center of the (*m,n*)-indexed cell-cluster in the Cartesian coordinates as indicated in (2). Consequently, multiplying *f*(*x, y*) by *Comb* function^45^, gives a discrete function (*f*_1*s*_(*x*_*m*_*, y*_*n*_)) as shown in (3) under the assumption of infinite dimensions of the reflectarray plane surface. For the bounded reflector plane (with area *L* × *L*), (3) should be multiplied by $Rect$ function shown in (4). The reflectarray composed of cell-clusters indicated in Fig. 3, schematically. Each square represents a cell-cluster with the relevant *m,n* indices.

2 $$x_{m} = \frac{{\left( {2m - 1} \right)a}}{2}, y_{n} = \left( {2n - 1} \right)a/2$$

3 $$f_{1s} \left( {x_{m} ,y_{n} } \right) = f\left( {x,y} \right).comb\left( {x/a + 0.5,y/a + 0.5} \right)$$

4 $$f_{2s} \left( {x_{m} ,y_{n} } \right) = f\left( {x,y} \right).comb\left( {x/a + 0.5,y/a + 0.5} \right) .Rect\left( {x/L,y/L} \right)$$

To connect the sampled function $f_{2s} \left( {x_{m} ,y_{n} } \right)$ to the tangential field above cell-clusters,(4) should be convolved to the $Rect$ function, of which the dimensions are proportional to the dimension of cell-cluster (*a* × *a*). Consequently, the specified tangential electric field distribution on the reflectarray composed of cell-clusters is obtained as follows:

5 $$e\left( {x,y} \right) = {\varvec{f}}_{{2{\varvec{s}}}} *Rect\left( {x/a ,y/a} \right)$$

The related far-field pattern may be calculated using the Fourier optics method. The Fourier transform of $e\left( {x,y} \right)$ is *E*(*u, v*) as given in (6). This formula shows the far-field distribution, obtained from the specified reflectarray structure composed of cell-clusters. For the comparison of *F(u,v)*, which is considered as the desired far-field pattern, at the start of the design, and ${\text{E}}\left( {{\text{u}},{\text{v}}} \right)$ as the created far-field pattern from the designed reflectarray structure, it is required to analyse (6) accurately.

6 $$E\left( {u,v} \right) = \left\{ {F\left( {u,v} \right)*\left( {1/a^{2} } \right)comb\left( {\left( {u - m\pi a} \right),\left( {v - n\pi a} \right)} \right) \sin c\left( {ua/2} \right)\sin c \left( {va/2} \right)} \right\}\,*\,a^{2} \sin c\left( {Lu/2} \right)\sin c\left( {Lv/2} \right)$$

As observed in (6), ${\text{E}}\left( {{\text{u}},{\text{v}}} \right)$ is a function of ${\text{F}}\left( {{\text{u}},{\text{v}}} \right)$, where ${\text{F}}$ is convolved and multiplied by *Comb* and *Sinc* functions that are related to the cell-cluster and reflectarray dimensions. Consequently ${\text{E}}$ is obtained from ${\text{F}}$ and also as a function of *a* and *L*, in which *a* indicates the cell-cluster dimension, and *L* is the total length of reflectarray.

For a more detailed study, it is seen in (6) that the parameter “*a*” appears in the *Comb* function argument, and also in the *Sinc* function, which affects the far-field pattern. In sub-sections “The effect of cell-cluster dimensions on far-field pattern” and “The effect of cell-cluster dimension on the generated pattern periodicity and filtering mechanism”, these effects will be explained comprehensively. The term $L$ related to the total reflector dimensions, can be seen in *Sinc (sin(πx)/(πx))* function argument, placed at the end of (6). This parameter causes oscillating ripples to appear in the far-zone pattern. The number of these ripples is proportional to the total length of structure. This phenomenon can be proved by Woodward-Lawson theorem^44^.

### The effect of cell-cluster dimensions on far-field pattern

As seen in (6), the coefficient *sinc*(*ua/*2)*sinc*(*va/*2) imposes some degradation on the magnitude of pattern leading to some deviation from the desired pattern (*F*(*u, v*)), which is proportional to parameter *a*. Figure 3 shows the magnitude of fluctuations of a one-dimensional *Sinc* function for different values of cell-cluster dimensions (compared to the wavelength). Observe that by increasing *a*, its amplitude decreases significantly in the region closer to the sides. Consequently, this factor should be considered for the selection of cell-cluster dimensions.

### The effect of cell-cluster dimension on the generated pattern periodicity and filtering mechanism

**Figure 4:**
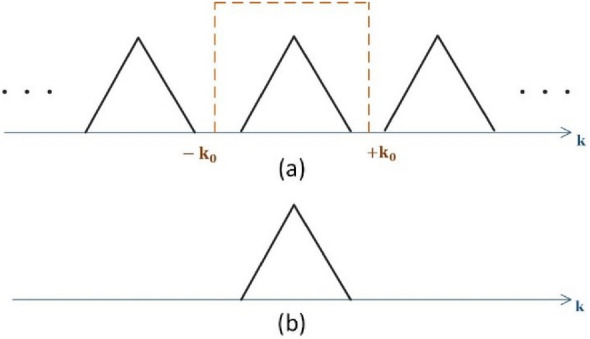
Filtering mechanism of the pattern function around the visible region. (**a**) Periodic function of *F* (*u, v*) and the filter position. (**b**) Regenerated function after filtering.

Considering (6), the convolution of *F*(*u, v*) with the *Comb* function, leads to the periodic repetition of *F*(*u, v*). For the construction of original function, it is necessary to perform a filtering operation on the function *E*(*u, v*). Note that the period of *E*(*u, v*) should be selected in such a way that the adjacent functions do not overlap (as shown in Fig. 4). This periodicity is proportional to the cell-cluster dimension. According to the Nyquist theorem, to avoid overlapping terms, the dimension of cell-cluster must satisfy *a* < *λ/*2 (*λ* is the free-space wavelength).

As mentioned in the previous section, the corresponding variables for far-field patterns have been defined as *u* = *k*0 sin *θ* cos *ϕ* and *v* = *k*0 sin *θ* sin *ϕ*, where *θ* and *ϕ* indicate the spatial spherical angles. Therefore, in the visible region, the spectral variables *u* and *v* change in the interval [*− k*0*, k*0]. As shown in Fig. 4, the required filter width for the rejection of unwanted band should be equal to 2*k*0. This width is exactly equal to the width of the visible region^44^. Outside this interval unwanted periods will be physically removed. Therefore, in practice, there is no need to design a band-pass filter.

### Selecting an optimum dimension for the cell-cluster

As mentioned in two previous sections, to reduce the impact of cell-cluster dimension on *F*(*u, v*) interference, they should be selected smaller than *λ/*2, and to reduce the impact of *Sinc* function on the pattern declination, the cell cluster dimension “*a”* should be considered as small as possible. On the other hand, the increase of the number of cells in the cell-cluster of reflectarray configuration, leads to reduction of phase error and electrical bias circuits. This characteristic calls for the design of cell-cluster dimension to be as large as possible. So, for a suitable design, a trade-off should be considered, commensurate to the demands and available possibilities.

## Design of graphene-based reflectarray using cell-cluster

So far, in this paper, the implementation of cell-cluster in the reflectarray design, and its impact on the far-field pattern have been investigated theoretically. Also, the related mathematical formulations are derived. To validate the proposed method, two examples are designed in this section. Both of them are reflectarrays composed of cell-clusters. The CST full wave simulation software is used to investigate the effect of cell-cluster dimensions on the far field pattern in each proposed design. In the first example (section “Design of reflectarray using surface impedance cells generating flat-top shaped pattern”), cell-clusters are substituted by the surface impedance type material in the CST simulator. The parametric values of each unit are set to give a flat-top pattern in the far-field radiation. In the second example in sections “Design of reflective metasurface for generation of pencil-beam at different angles and Generation of flexible directive beams”, a reflectarray composed of cell-clusters is designed. In this structure, the cell-clusters are composed of graphene unit-cells. In each cell-cluster all unit-cells have identical electrical biases, and all the patches have the same physical size, to generate the same reflection characteristics in each cell-cluster. The full structure is designed to generate a pencil-beam in a desired direction. Since in this paper the effect of cell-cluster dimension is of interest, in sections Design of reflectarray using surface impedance cells generating flat-top shaped pattern and Generation of flexible directive beams, different cell-cluster dimensions are implemented in the reflectarray to examine their effects separately on the far-field pattern. Optimum dimensions are selected for the designs.

### Design of reflectarray using surface impedance cells generating flat-top shaped pattern

As mentioned before, in this paper the goal is to design and examine the impact of cell-cluster dimensions on the far-field pattern. Here, a one-dimensional flat-top pattern is designed using the above-mentioned analytical formulations and design procedure. The aperture field is assumed to vary in the x-direction and the field along the y-direction is uniform. To obtain a flat-top pattern, it is convenient to produce a *Sinc* function as an aperture field distribution. In this section, we use the surface impedance material for each cell-cluster to generate an ideal uniform field on each unit. (This model is analogous to the *Rect* function in (4)). In order to define the aperture field distribution on the reflectarray structure, $f\left( {x,y} \right)$ should be substituted by *Sinc* function in (4), (5). Similarly, the parameter ${}^{^{\prime\prime}}a^{^{\prime\prime}}$ is substituted by the cell-cluster dimension, and ${}^{^{\prime\prime}}L^{^{\prime\prime}}$ is substituted by the reflactarray length. To demonstrate a one-dimensional flat top pattern, the aperture-field variation should be only in one direction (x-direction here), so the equation has one variable *x* only, and the *y* component should be eliminated. Thus (5) changes to:

7 $$e\left( x \right) = sinc\left( {2\sin \left( {\theta_{0} } \right)x/\lambda_{0} } \right).comb\left( {x/a + 0.5} \right) .Rect\left( {x/L} \right)*Rect\left( {x/a } \right)$$

where $\theta_{0}$ is the desired direction of the pencil-beam in the far-field radiation. In this model, $e\left( x \right)$ is considered as the tangential electric field on the reflector surface as an aperture field distribution. As seen in (7), $e\left( x \right)$ is a purely real function. In order to produce such a field on the reflector surface, we use the “surface impedance model” instead of cell-clusters, with the equivalent related magnitude.

The input impedance of each unit, can be calculated using (8). Since the illumination source, is a uniform normal plane wave, ${\Gamma }\left( {\text{x}} \right)$ can be replace with $e\left( x \right)$ (the desired aperture field distribution). Since, each unit is considered as a nontransparent element, $\eta_{in} \left( x \right)$ can be replaced with $\eta_{sheet} \left( x \right)$, in which, $\eta_{sheet}$ is equal to related surface impedance, and also considered as a nontransparent material^46^. implementing $\eta_{sheet} \left( x \right)$ (calculated from (8) and related $e\left( x \right)$), and using a plane wave incident source, the desired aperture field distribution created on the reflectarray surface, and the desired far-field pattern is obtained.

8 $$\eta_{in} \left( x \right) = \eta_{0} \frac{{1 + {\Gamma }\left( {\text{x}} \right)}}{{1 - {\Gamma }\left( {\text{x}} \right)}}$$

**Figure. 5:**
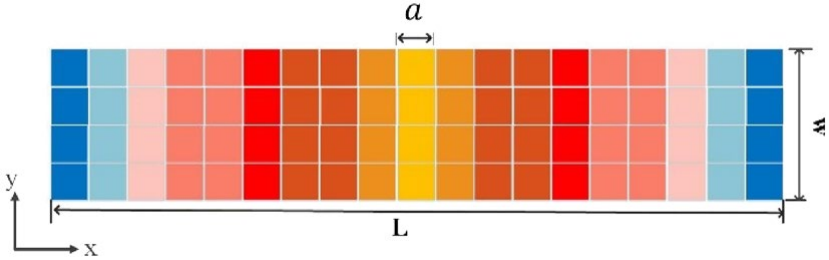
Impedance distribution of the proposed reflectarray for the generation of flat-top pattern.

**Figure 6:**
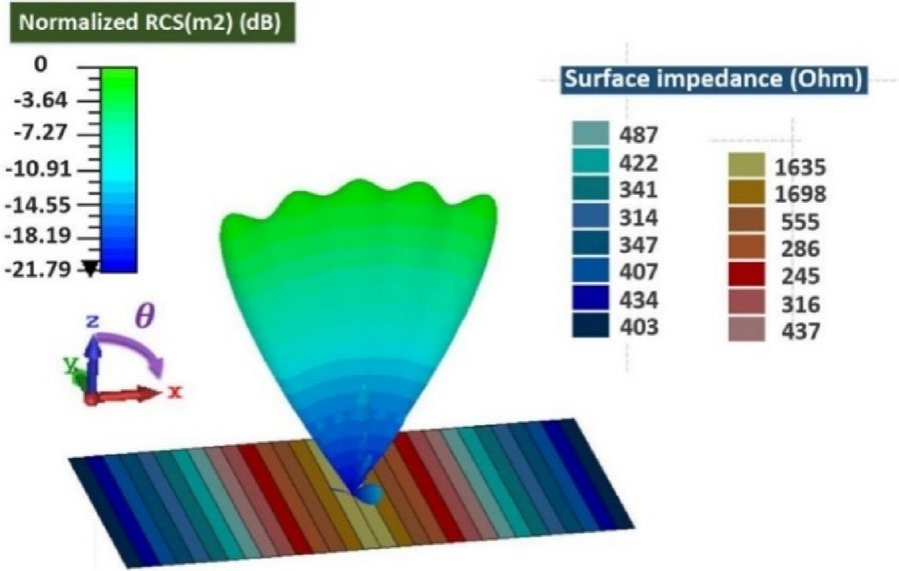
Generation of one-dimensional flat-top pattern in the elevation direction using surface impedance ribbons.

**Figure 7:**
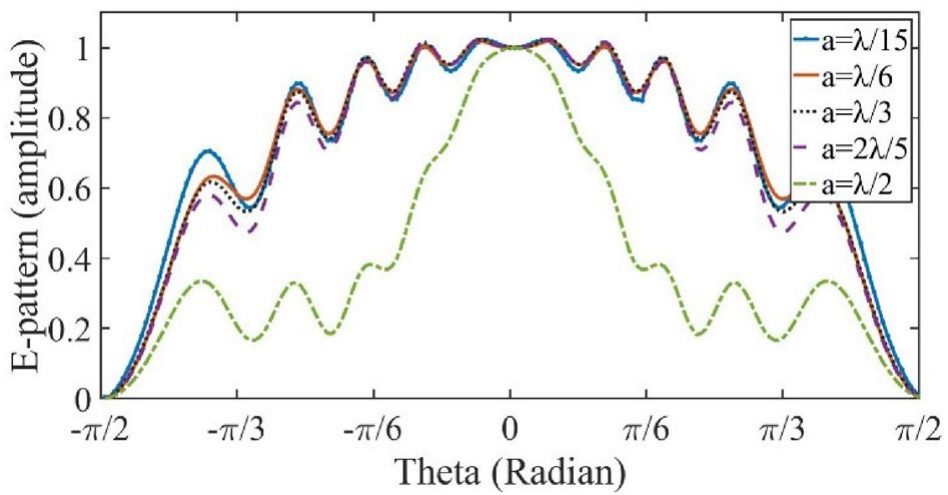
The flat-top pattern amplitude for different values of cell-cluster dimensions.

**Figure 8:**
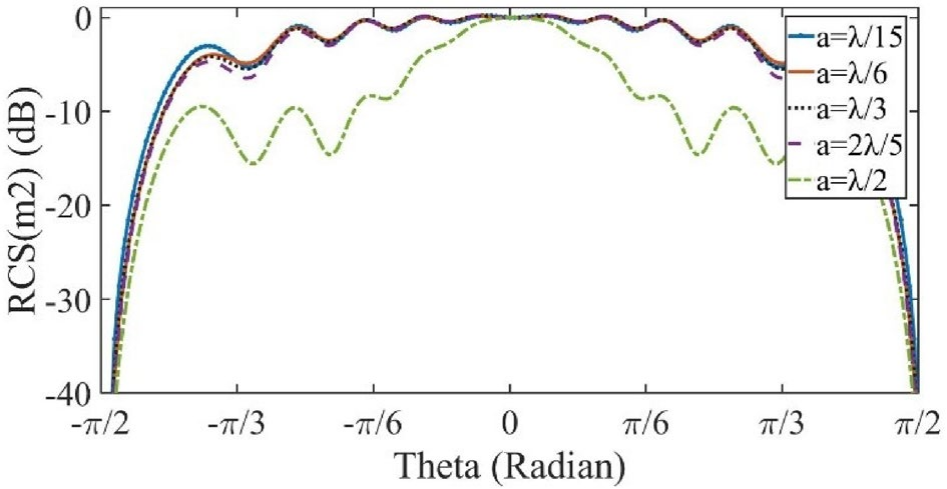
The flat-top pattern in dB for different values of cell-cluster dimensions.

The simulation results are obtained by the transient solver in the CST Studio software. As mentioned before, the surface impedance boundary condition is used to realize the proposed metasurface. Figure 5 shows the implemented surface impedance. Each color represents a cell-cluster with a certain value of reactance. The specified objective pattern is assumed to be a flat-top pattern with beam-width of *π/*3 radians at operating frequency f = 1 THz. The geometrical dimensions *L*, *W* and *a* are selected as 3000, 1500, and 90 µm, respectively. Figure 6 shows the simulation results of the reflected pattern in the case of perpendicular plane-wave illumination as the excitation. In this figure, the rectangular cells with surface impedances in the range of 314 to1635 Ω are implemented. The width of each cell, in the *x-*direction, is 0*.*3*λ*. In another example, the beam-width is selected as 0*.*82*π,* in order to see the effect of cell-cluster dimensions more clearly. We have no variation in the *y*-direction. So, the magnitude of surface wave on the reflector surface, varies only in the *x*-direction. In Figs. 7 and 8, the far-field patterns for different values of “*a”* are plotted. Observe in Fig. 7 that by increasing the dimensions of the cell-cluster, the main beam drops significantly at *θ* = *π/*3. In this simulation example, in order to obtain a flat-top pattern with the beam declination less than 0.6, it is necessary to select the cell-cluster dimensions smaller than *λ/*3.

### Design of reflective metasurface for generation of pencil-beam at different angles

In order to create a pencil-beam in the far-field region in the desired direction ($\theta_{0}$), a phase gradient as an aperture-field distribution should be created. For this purpose, $f\left( {x,y} \right)$ should be substituted by $exp\left( { - ik_{0} \sin \theta_{0} x} \right)$ in (4,5) to obtain (9).

9 $$e\left( x \right) = exp\left( { - ik_{0} \sin \theta_{0} x} \right).comb\left( {\left( {x + 0.5} \right)/a} \right) .Rect\left( {x/L} \right)*Rect\left( {x/a } \right)$$

As seen in (9), $e\left( x \right)$ is a phase-only variable function. Accordingly, phase-only impedance variable unit-cells are required. Metal patch unit-cells, with phase-only variation in the surface impedance ($Z_{t}$), are commonly employed. However, since in this paper the goal is to design reflectarrays using graphene cell-clusters, it is required to design the graphene unit-cell through phase-only variation in its reflected field.

**Figure 9:**
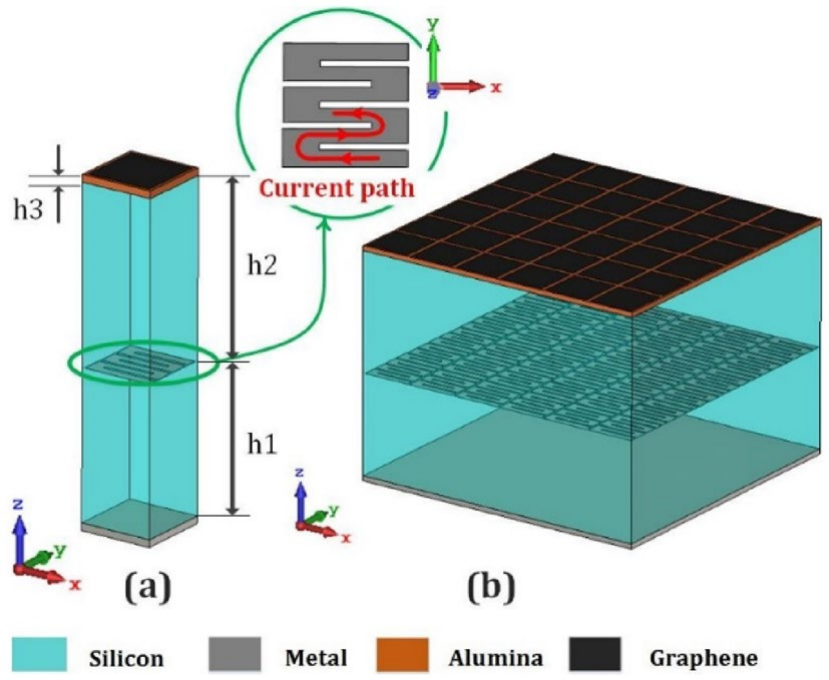
(**a**) The proposed unit-cell composed of two layers, the middle layer is a metallic patch located on the Silicon layer (*h*1 = 28* µm*) as a dielectric spacer, and the top layer is graphene patch located on Alumina layer (*h*3 = 0*.*2* µm*). Another Silicon layer (*h*2 = 32* µm*) is located between the Alumina and metal slot layer. (**b**)The cell-cluster composed of 6 × 6 unit-cells.

**Figure 10:**
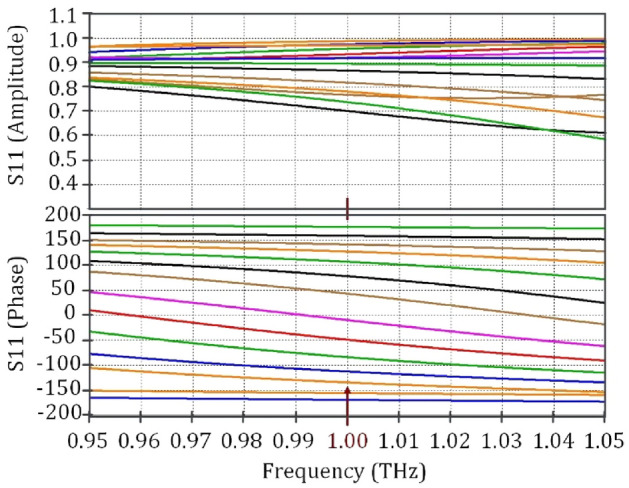
Amplitude and phase variations of the proposed unit-cell for different voltage biases and slot lengths.GeapheneFermi level is 0 to 0.2 eV, and the relaxation time is 1 ns.

In this section, the graphene-based reflectarray structure is designed to generate flexible pencil-beams. Multilayer graphene-based cell-clusters are exploited as the constituent elements to achieve our required phase variations. To attain an appropriate characteristics, the proposed unit-cell should satisfy two criteria: (1) The phase variation interval should cover 360 degrees; and (2) The magnitude variations should be kept as low as possible. (The acceptable range is 0.3 dB.) Fig. 9 shows the proposed unit-cell configuration and the corresponding cell-cluster consisting of 36 elements. As shown in Fig. 9a, the unit-cell is composed of a graphene patch (top layer), a slotted metallic patch (middle layer), and a metallic reflector (bottom layer). Two dielectric spacers with *ϵr* = 11*.*9, and thicknesses of 28 and 32 µm are used as top and bottom host media, respectively. The slotted metallic patches in the middle layer, create a spiral metallic track, in which the length of the induced current paths can be adjusted. The phase-change steps are tuned by changing the bias voltage of the graphene patches and the middle-layer metal-slot lengths. The use of these degrees of freedom, allows us to minimize the undesired magnitude variations. Figure 10 shows the simulated reflection coefficients for different bias voltages, and metal slot lengths. The corresponding phase varies from − 180 to +180 degrees which meets our requirements. Observe also in Fig. 10, that another advantage of the proposed unit-cell over the single-layer counterparts^47^ is that the phase variation is less sensitive to the bias voltage, which facilitates the implementation process. The use of metal patch in the middle layer, causes the reduction of losses, compared with the structure made up of two layers of graphene patch. On the other hand, the chemical potential range decreases significantly (namely 0 to 0.2 $ev$).

### Generation of flexible directive beams

**Figure 11:**
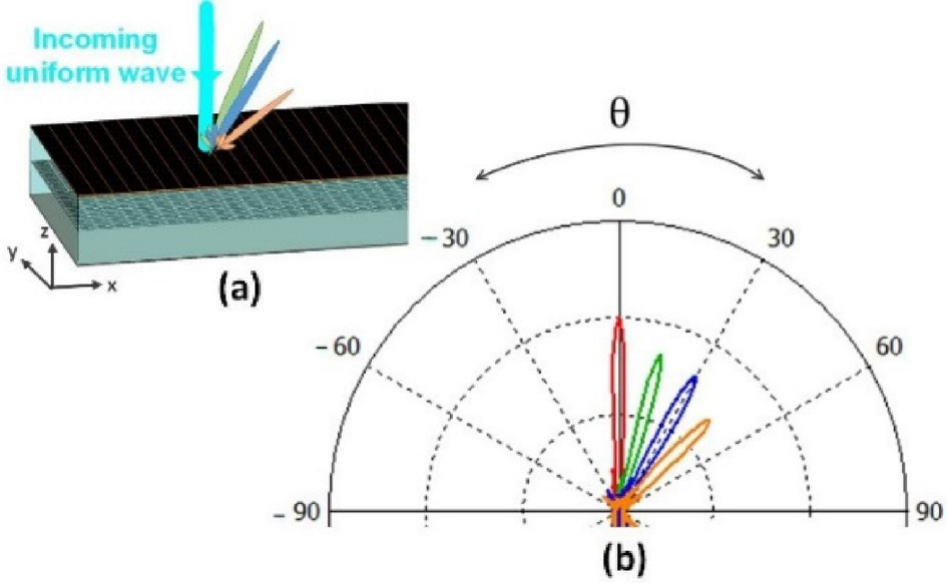
(**a**) Schematic of the proposed reflectarray forgenerating reconfigurable pencil-beams. (**b**)Simulation results for the radiation pattern at *ϕ* = 0*◦*.

**Figure 12:**
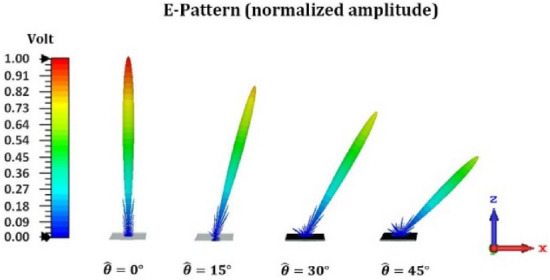
3D E-pattern of pencil-beam for different radiation angles ($\hat{\theta } = 0^\circ ,15^\circ ,30^\circ ,45^\circ \& \hat{\phi } = 0^\circ$).The dimension of cell-cluster is 9*9 unit-cells.

A one-dimensional array with dimensions of 10*λ* × 10*λ* and a phase variation along the x-direction is designed to generate a directive beam in the far-field pattern at a desired direction. The cell-cluster proposed in section "Design of reflective metasurface for generation of pencil-beam at different angles" is used here as reflective elements. In order to idealize the simulation process and eliminate the edge diffractions, the perfect electric (PEC) boundary condition is used on both sides of the edges as two planes perpendicular to the y-axis on both sides of reflector structure. The E-field of wave is $\hat{y}$-directed. In practical cases, an elimination of edge diffractions may be achieved by shaped-edge grounded bottom layer, such as slotted or serrated edges^48,49^. The simulation results of the proposed structure for perpendicular illumination are shown in Fig. 11. The far-field radiation patterns are directed at 0, 15, 30 and 45 degrees. Observe in Fig. 11 that as the reflection angle increases, the far-field amplitude decreases (in the 2D-pattern). Also Fig. 12 shows the related 3D pattern.

**Figure 13:**
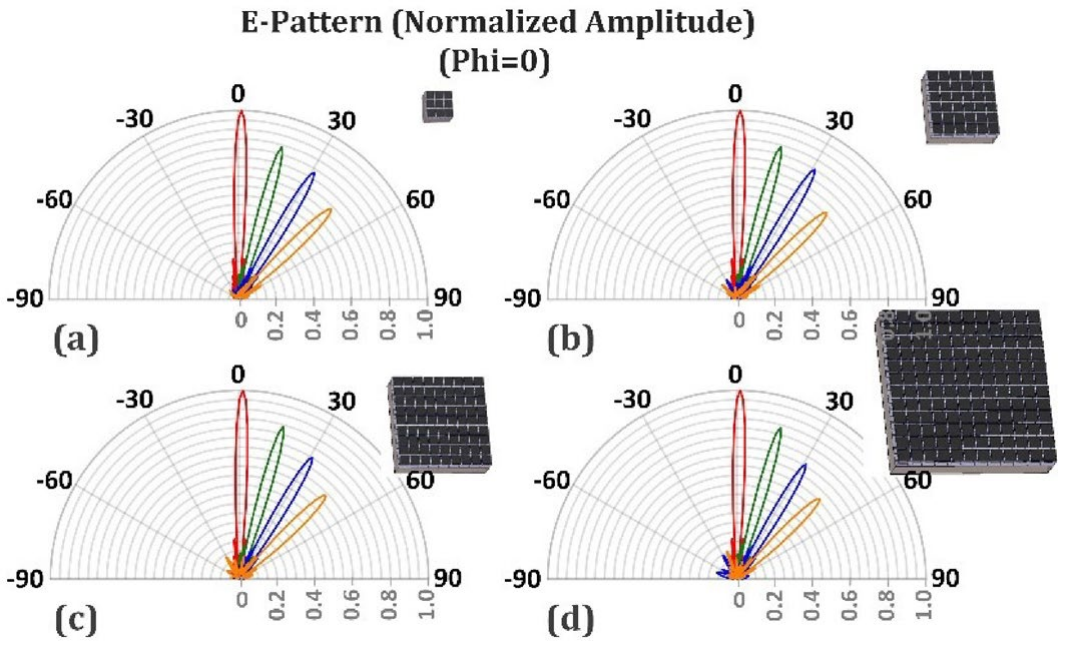
Variation of the radiated pencil beam for different reflectarrays composed of different cell-cluster dimensions. (**a**) Cell-cluster composed of 3 × 3 unit-cells. (**b**) Cell-cluster composed of 6 × 6 unit-cells. (**c**) Cell-cluster composed of 9 × 9 unit-cells. (**d**) Cell-cluster composed of 12 × 12 unit-cells. The operating frequency is supposed to be 1 THz and each cell-cluster composed of graphene unit cells with dimensions of 10 × 10* µm*.

One reason for the reduction of directivity is the amplitude ripples in the far-field generated due to the use of cell-clusters with specific dimensions, as mentioned in Section “The effect of cell-cluster dimensions on far-field pattern”. To compare the effect of cell-clusters dimensions on the directivity, the simulation results for various dimensions of the cell-cluster are plotted in Fig. 13. Another reason for the above-mentioned directivity drop, is the space wave conversion into the surface wave, and power dissipation due to the surface wave propagation on the reflector surface. Generally, in reflectarrays, with the increase of the reflection angle, some power may be converted to the surface wave, leading to the reduction of the amplitude of the main lobe, while the side lobe level increases^50–52^. Due to the power loss effect in graphene patches, the side lobes do not increase significantly. Observe in Fig. 13 that by the increase of reflection angle, the reduction of main lobe level dominates and its decrease due to the cell-cluster enlargement is not appreciable.

**Figure 14:**
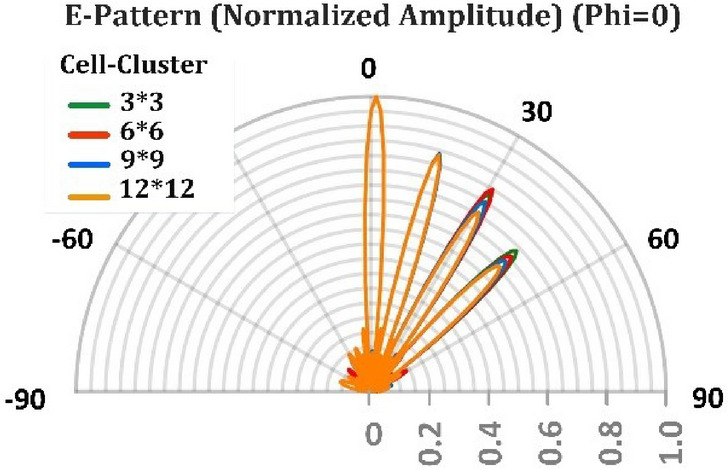
Variation of beam direction in four angels of $0^\circ , 15^\circ , 30^\circ , 45^\circ$, for different cell-cluster dimension.

Table. 1 compares the radiation characteristics of the proposed reflectarray for different cell-cluster dimensions. In Fig. 14 the four modes of Fig. 13a,b,c,d are plotted together, so that the reduction due to cell-cluster enlargement is clearer. Table 2 compares the results obtained in this work with other graphene-based reflectarrays generating pencil-shaped beams. In our work the amplitude variation of unit-cell is less than 3 dB and the phase-variation range is 360 degrees.

**Table 1:** Radiation characteristics of the proposed antenna for different values of cell-cluster dimensions.

No	Cell-cluster dimension	Side lobe level in *ϕ* = 0*◦* plane (dB)	Angular beamwidth in *ϕ* = 0*◦* plane (deg)	Directivity (dBi)	Efficiency (%)
**Main-lobe-direction: 0Deg**					
1	3*3	− 13.3	5.2	32.1	91.6
2	6*6	− 13.3	5.2	32.1	91.6
3	9*9	− 13.3	5.2	32.1	91.6
4	12*12	− 13.3	5.2	32.1	91.6
**Main-lobe-direction: 15Deg**					
5	3*3	− 13.5	5.3	32.0	85.6
6	6*6	− 13.6	5.3	32.0	85.4
7	9*9	− 13.6	5.3	32.0	85.7
8	12*12	− 12.7	5.3	32.0	84.9
**Main-lobe-direction: 30Deg**					
9	3*3	− 12.9	6	31.5	85.1
10	6*6	− 12.9	6	31.5	86.1
11	9*9	− 13.2	6	31.5	82.7
12	12*12	− 12.2	6	31.5	82.0
**Main-lobe-direction: 45Deg**					
13	3*3	− 12.9	7.2	30.5	81.9
14	6*6	− 12.6	7.2	30.5	80.7
15	9*9	− 12.8	7.2	30.5	80.5
16	12*12	− 11.9	7.4	30.5	77.7

**Table 2:** Performance comparison of the proposed antenna and other grapheme based reflectarrays.

	Working frequency (THz)	Gain (dB)	Reflectarray dimension	Unit cell dimension	Unit-cell phase variation (Deg)	Unit cell amplitude variation (dB)
Carrasco and Carrier^13^	1.3	26.9	2500 unit-cells	λ0/16	290	0 to − 7.5
Li et al.^53^	1	19.2	11*11 unit-cells	λ0/3	300	0 to − 18
Hassan et al.^40^	1.6	19	20λ*20λ	λ0/2	525	− 1 to − 13
our	1	29.4	10λ*10λ	λ0/10	360	0 to − 3

### Reflectarray bias circuits

**Figure 15:**
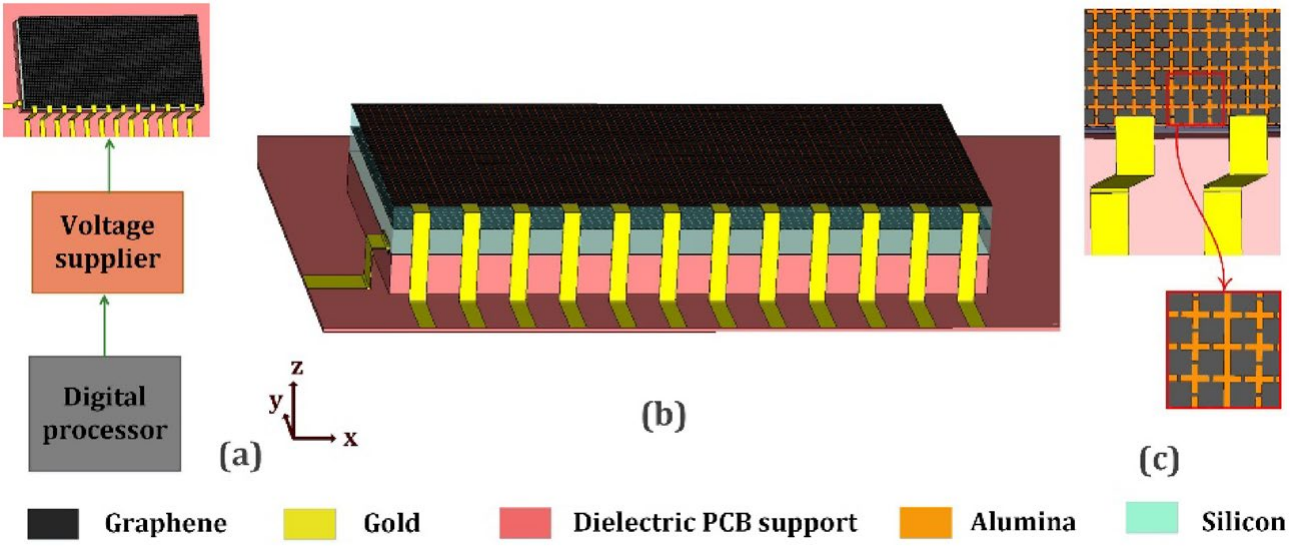
Schematic of reflectarray with bias circuit. (**a**) schematic of bias voltage program and supplier. (**b**) unit cell electrical connection in each cell cluster. (**c**) reflectarray schematic.

As mentioned before, in our design, for changing the chemical potential of graphene unit-cells, the variable electric voltage bias should be applied. For this purpose, a metal voltage connector should be connected to the graphene patches directly. As shown in Fig. 15a, gold electric connectors are implemented for this purpose. A digital processor is implemented, for the programming of the required bias voltage for each cell-cluster. The digital processor is connected to a voltage supplier to produce the required biasing voltage amplitude. As mentioned, each cell-cluster is connected to a single bias voltage. For this purpose, in each cell-cluster, all graphene patches, are connected to each other (Fig. 15b). The whole reflectarray structure is located on a single flat dielectric, as the PCB holder (Fig. 15c). A zero (or ground voltage) is connected to a grounded metal layer, under the reflectarray, as shown in Fig. 15.

## Conclusion

An architecture of graphene cell-clusters composed of equivalent graphene unit-cells is proposed for the realization of reflectarrays. The performances of the graphene unit-cell and cell-cluster reflectarrays are investigated, and appropriate design procedures are developed for them. The application of graphene cell-cluster reflectarrays, has several advantages, such as elimination of phase error inherent in unit-cell implementations, and simplification of related electronic circuitry in tunable structures. The methods of Fourier-optics and aperture field estimation are applied to study the effect of cell-cluster dimensions on the far-field pattern. The optimum dimensions of the graphene cell-cluster for the reflectarray are determined for the related examples. For the assessment of proposed methods, a reflectarray composed of surface impedance model is designed to produce flat top far-field pattern. Finally, a reflectarray composed of graphene cell-clusters for different reflection angles is designed, and the effects of cell-cluster dimensions are investigated and compared.

## Data Availability

The data that support the findings of this study are available from ParinazHosseini but restrictions apply to the availability of these data, which were used under license for the current study, and so are not publicly available. Data are however available from the authors upon reasonable request and with permission of ParinazHosseini.
